# Trends in MD-PhD representation and match outcomes across competitive specialties, 2007–2024

**DOI:** 10.1371/journal.pone.0357960

**Published:** 2026-09-10

**Authors:** Albert E. Zhou, Timothy Klufas, Erik Choi, Yee Won Kim, Jenny J. Kim, Akua Sarfo, Brett Sloan, Hao Feng

**Affiliations:** 1 Department of Dermatology, UConn Health, Farmington, Connecticut, United States of America; 2 School of Medicine, New York Medical College, Valhalla, New York, United States of America; 3 School of Medicine, University of Connecticut, Farmington, Connecticut, United States of America; 4 Department of Dermatology, University of California – Los Angeles, Los Angeles, California, United States of America; Shiraz University of Medical Sciences, IRAN, ISLAMIC REPUBLIC OF

## Abstract

Physician-scientists trained through MD-PhD programs play important roles in translational research. Despite the growing emphasis on research in residency applications, it remains unclear whether competitive specialties demonstrate a preference for MD-PhD graduates. This study evaluates differences in representation and match outcomes between MD-PhD and MD applicants across select specialties from 2007–2024, as well as the influence of specialty-level compensation and structural research infrastructure on outcomes. We analyzed data from the Association of American Medical Colleges (2015–2023), the National MD-PhD Program Outcomes Study (1975–2024), and the National Resident Matching Program’s Charting Outcomes in the Match™ (2014–2024). Chi-square tests compared match rates between MD-PhD and all U.S. MD senior applicants across dermatology, orthopedic surgery, neurosurgery, otolaryngology, plastic surgery, and diagnostic radiology. Negative binomial models measured changes in MD-PhD resident representation. Spearman rank correlations assessed associations between specialty-level physician compensation, NIH departmental funding, number of dedicated Physician-Scientist Training Programs, and MD-PhD outcomes. From 2015–2023, MD-PhD representation among residents remained stable with match rates varying by specialty and year. Dermatology and diagnostic radiology demonstrated strong match outcomes for MD-PhDs, whereas orthopedic surgery showed declining success for MD-PhD applicants. Neurosurgery, otolaryngology, and plastic surgery,had variable or inconsistent trends. Compensation and NIH funding were not significantly correlated with MD-PhD representation or match advantage. Physician-Scientist Training Programs availability showed the strongest associations with both outcomes but was not statistically significant given the small sample size. MD-PhD training does not consistently confer a match advantage in competitive specialties. The availability of research training infrastructure, compensation, and NIH funding may influence MD-PhD match success. Additionally, residency program preferences, such as clinical metrics, as well as MD-PhD applicant preferences may both affect match outcomes. Enhanced mentorship, specialty-specific exposure, and financial support may help support the physician-scientist pipeline across specialties.

## Introduction

Since their creation in the 1950s, MD-PhD programs have trained physician-scientists, who harbor unique perspectives in translational research, bridging bench-to-bedside scientific principles [1]. Matriculants to MD-PhD programs typically display academic excellence with strong undergraduate grade points averages (GPAs), Medical College Admissions Test (MCAT) scores, and extensive research experiences [2]. MD-PhD programs integrate the complementary strengths of MD and PhD programs. Standard MD programs prioritize diagnosing and treating patients, with a four-year education oriented toward clinical reasoning, patient care, and procedural skills. While MD candidates often participate in research, their primary role is rooted in patient care. In contrast, PhD programs are five- to eight-years long and devoted to producing independent scientists. PhD candidates do not receive clinical training and are focused on research and the generation of new knowledge. The MD-PhD pathway integrates the training of these programs, combining clinical practice with research over seven to eight years. MD-PhD graduates are prepared to pursue careers as physician-scientists [2] who play vital roles in converting scientific discoveries into practical clinical applications that improve patient care [3].

Historically, the factors influencing match rates have included United States Medical Licensing Examination (USMLE) Step 1 and 2 scores, research productivity, program signaling, geographic preference, letters of recommendation (LORs), personal statements, and clerkship grades [4]. With the transition of USMLE Step 1 to pass/fail in 2022, research productivity, along with other metrics, have grown in importance for residency applicants, potentially demonstrated by the increasing number of medical students pursuing research years [5–8]. MD-PhD graduates, who possess comprehensive scientific training, represent a distinct subset of applicants, but it remains unclear whether their research background confers a measurable advantage in the residency match process.

Several initiatives specifically designed to support the training of physician scientists exist. The Medical Scientist Training Program (MSTP) is an MD-PhD program supported by the NIH through T32 grants [9]. Physician Scientist Training Programs (PSTP) are postgraduate training programs with the goal of providing protected research time during residency and fellowship training to facilitate the transition period between the completion of an MD-PhD, DO, or MD degree and an applicant’s first faculty position. Aspiring physician-scientists can apply to specific research tracks within clinical specialty programs, best exemplified by the American Board of Internal Medicine Research Pathway and the American Board of Pediatrics Integrated Research Pathway [10]. 2 + 2 tracks, similar to PSTPs, are another formal research track for residents where one commits to two years of clinical residency training followed by two years of mentored research training with the goal of preserving allocated research time [11]. Programs such as the Intramural NIH Clinical Research Training Program provide additional mentorship and opportunities for aspiring physician-scientists [12].

Here, we evaluate whether MD-PhD graduates have improved match rates in competitive specialties where research productivity is a primary selection criterion. Dermatology, orthopedic surgery, neurosurgery, otolaryngology, plastic surgery, and diagnostic radiology were selected based on historical competitiveness, as measured by USMLE Step 2 clinical knowledge (CK) score, research productivity, match rate of applicants into each specialty, the percent of matched students that were Alpha Omega Alpha (AOA) honors society members, and positions-to-applicant ratios [8,13]. The six selected specialties have been consistently recognized as highly selective across these multiple metrics within the study period of 2007–2024, despite fluctuations in match rates and applicant-to-position ratios [13]. Specialties utilizing separate match systems (*i.e.,:* San Francisco Match), such as urology and ophthalmology were excluded as MD-PhD applicant-level data were not captured in the NRMP Charting Outcomes™ dataset used for this analysis. Analysis was conducted using data from the National Resident Matching Program (NRMP), Association of American Medical Colleges (AAMC), and the National MD-PhD Program Outcomes Study [14,15].

In this study, we tested three hypotheses: 1) MD-PhD training confers a statistically significant match advantage over MD-only applicants across the competitive specialties studied, 2) specialty-level clinical compensation would be inversely associated with MD-PhD resident representation, and 3) higher specialty-level compensation would correlate with a smaller MD-PhD match advantage. The latter hypotheses were motivated by the structural disincentive created by NIH salary cap constraints in high-earning fields, potentially discouraging both recruitment and retention of MD-PhD graduates.

## Methods

Data on MD-PhD graduates were obtained from the AAMC Report on Residents, from 2015−2023, and the National MD-PhD Program Outcomes Study, which reports data from 1975–2024, and the NRMP Charting Outcomes in the Match™ from 2014−2024 [8,15–17]. The annual AAMC Report on Residents outlines the number of active residents in each specialty. The total number of active residents and the number of active residents holding MD-PhDs was retrieved from Table B4, “MD-PhD Residents, by Graduate Medical Education (GME) Specialty.” The percentage of MD-PhD residents in different specialties was retrieved from the National MD-PhD Program Outcomes Study, which illustrates the representation of MD-PhD residents across different specialties [15]. Within the NRMP datasets, the proportion of matched applicants holding a PhD was obtained from Chart DM-9 (“Other Characteristics of U.S. Seniors”).

The percentage of matched MD-PhD applicants was calculated by dividing the number of applicants matched over the total number of applicants in each report. The overall percentage of matched U.S. MD senior applicants was calculated from Table 1 of the NRMP Charting Outcomes in the Match™ by dividing the number of U.S. MD senior matched applicants by the total number of U.S. MD senior applicants [18]. Of note, the match data from the NRMP does not distinguish between Physician-Scientist Training Programs (PSTPs) and non-PSTP program applicants.

**Table 1:** Percentage of MD-PhD residents in three most overrepresented specialties (blue), three most underrepresented specialties (orange), three specialties with the greatest number of residents (green), and “research-oriented” specialties (grey), ranked from highest to lowest percentage, 2014. Data adopted from 2018 MD-PhD Outcomes Study.

Specialty	Total Residents	Percentage of All Residents	MD-PhD Residents	Percentage of MD-PhD Residents
Medical Genetics	76	0.1%	14	18.4%
Radiation Oncology	678	0.9%	114	16.8%
Pathology: Anatomic and clinical	1,543	2.0%	220	14.3%
Neurosurgery	1,199	1.5%	98	8.2%
Dermatology	1,276	1.6%	76	6.0%
Plastic Surgery: Integrated	554	0.7%	27	4.9%
Internal Medicine	17,354	22.4%	731	4.2%
Pediatrics	8,040	10.4%	289	3.6%
Diagnostic Radiology	4,151	5.4%	119	2.9%
Otolaryngology	1,484	1.9%	32	2.2%
Surgery: General	6,912	8.9%	114	1.6%
Orthopedic Surgery	3,748	4.8%	36	1.0%
Thoracic Surgery	154	0.2%	1	0.6%
Plastic surgery: Non-integrated	309	0.4%	2	0.6%
Family Medicine	5,019	6.5%	26	0.5%
Total residents	77,461	_—	2,575	—

To explore factors associated with MD-PhD outcomes, specialty-level mean physician compensation, total NIH departmental funding, and the number of dedicated PSTPs were obtained for each specialty from the Doximity 2024 Physician Compensation Report, the Blue Ridge Institute for Medical Research (BRIMR) 2024 rankings, and the American Physician Scientists Association (APSA) program directory, respectively [19–21]. Correlations between each variable and the proportion of MD-PhD residents in 2014 as well as the mean MD-PhD match rate advantage were observed.

### Statistics

Chi-square tests were used to assess differences between U.S. senior MD applicants compared to MD-PhD applicants in the NRMP’s match outcome (*Charting Outcomes in the Match™)* from 2007–2024 (Stata, StataCorp, 2025). Negative binomial models were used to calculate annual changes in MD-PhD resident representation across the selected specialty from 2015–2023 (Stata, StataCorp, 2025). Data were obtained from AAMC Table B4. A *p-*value less than 0.05 was considered significant. Spearman rank correlations were calculated across the six specialties to assess associations between three specialty-level variables and two MD-PhD outcome measures. Specialty-level variables included mean physician compensation (Doximity, 2024), total NIH departmental funding (BRIMIR, 2024), and number of dedicated PSTPs per specialty (APSA program directory). Meanwhile, the MD-PhD outcome measures were MD-PhD resident representation (2014 National MD-PhD Program Outcomes Study) and mean MD-PhD match advantage, defined as the mean annual percentage-point difference between MD-PhD and overall applicant match rates across NRMP cycles from 2007–2024.

## Results

Representation of MD-PhD residents varied considerably by specialty. In 2013–2014, MD-PhDs constituted 3% of all residents nationwide [22]. During the same period, the highest proportions of MD-PhD residents were in medical genetics (18.4%, n = 14 of 76), radiation oncology (16.8%, n = 114 of 678), and pathology (14.3%, n = 220 of 1,543) (Table 1). By contrast, family medicine (0.5%, n = 26 of 5,019), non-integrated plastic surgery (0.6%, n = 2 of 309), and thoracic surgery (0.6%, n = 1 of 154) had the lowest representation. Among the specialties of focus in this study, neurosurgery (8.2%, n = 98 of 1,199), dermatology (6.0%, n = 76 of 1,276), and integrated plastic surgery (4.9%, n = 27 of 554) were relatively overrepresented, while diagnostic radiology (2.9%, n = 119 of 4,151), otolaryngology (2.2%, n = 32 of 1,484), and orthopedic surgery (1.0%, 36 of 3,748) had the smallest representation (Table 1). While internal medicine and pediatrics were the two specialties with the greatest number of total residents (17,354 and 8,040, respectively) and MD-PhD residents (731 and 289, respectively), both fields had a smaller proportion of MD-PhDs comparatively, with 4.2% (n = 731 of 17,354) of internal medicine and 3.6% (n = 289 of 8,040) of pediatrics residents holding MD-PhDs [15].

The proportion of MD-PhD graduates among active residents has stayed relatively stable across multiple specialties from 2015–2023 (Table 2). Negative binomial models revealed no statistically significant changes in year-to-year MD-PhD representation from 2015–2023 (2015–2021 for plastic surgery) (Table 2). In dermatology, the decrease in percentage of MD-PhD residents from 5.6% (n = 69 of 1,231) in 2015 to 3.9% (n = 54 of 1,397) in 2023 was not statistically significant (Table 2). Similarly, in orthopedic surgery, despite a decline in representation from 0.9% (n = 32 of 3,509) in 2015 to 0.8% (n = 29 of 3,720) in 2023, representation was also not statistically significant different. Diagnostic radiology had a slight reduction from 3.1% (n = 115 of 3,728) in 2015 to 2.9% (n = 93 of 3,205) in 2023. Plastic surgery showed the sharpest decline, from 3.2% (n = 30 of 936) in 2015 to just 0.4% (n = 4 of 1,124) in 2021. In contrast, neurosurgery increased from 8.8% (110 of 1,244) in 2015 to 9.2% (132 of 1,433) in 2023, while otolaryngology rose from 2.3% (n = 34 of 1,457) in 2015 to 2.5% (n = 42 of 1,680) in 2023 (Table 2).

**Table 2:** Annual number of MD-PhD and Total active dermatology, neurosurgery, orthopedic surgery, otolaryngology, plastic surgery, and diagnostic radiology residents, 2015–2023.

Year Range	2015	2016	2017	2018	2019	2020	2021	2022	2023	Annual percentage change	p-value
Dermatology MD-PhD Active Residents	69	63	59	65	61	54	69	79	54	−1.71%	0.894
Dermatology Total Active Residents	1231	1247	1254	1272	1294	1290	1365	1377	1397		
Neurosurgery MD-PhD Active Residents	110	105	106	121	124	122	123	122	132	+1.06%	0.960
Neurosurgery Total Active Residents	1244	1274	1281	1368	1382	1387	1409	1419	1433		
Orthopedic Surgery MD-PhD Active Residents	32	34	38	40	34	32	27	29	29	−0.73%	0.774
Orthopedic Surgery Total Active Residents	3509	3547	3592	3634	3645	3670	3703	3710	3720		
Otolaryngology MD-PhD Active Residents	34	34	33	34	33	32	38	42	42	−3.69%	0.936
Otolaryngology Total Active Residents	1457	1467	1487	1492	1527	1557	1607	1632	1680		
Plastic Surgery MD-PhD Active Residents	3	3	2	2	1	0	0			−29.23%	0.229
Plastic Surgery Total Active Residents	298	244	196	154	140	130	125				
Diagnostic Radiology MD-PhD Active Residents	115	105	92	88	84	86	91	89	93	+0.65%	0.955
Diagnostic Radiology Total Active Residents	3728	3509	3299	3247	3175	3169	3190	3215	3205		

Obtained from AAMC Table B4. MD‑PhD Residents, by GME Specialty. Annual changes in proportion of MD-PhD residents to all residents calculated using negative binomial models. Data on plastic surgery residents unavailable for 2022–2023.

**Fig 1:**
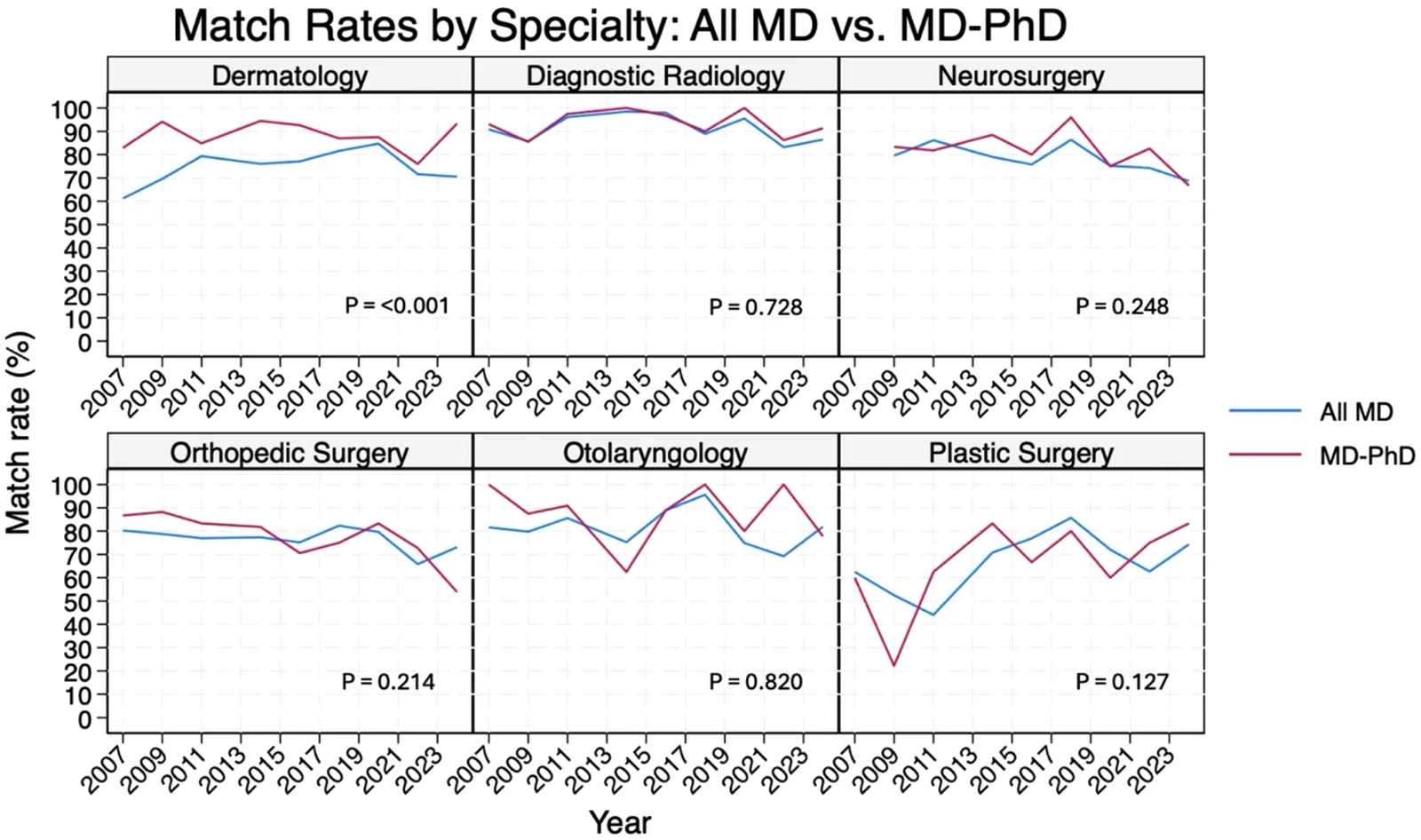
Match rate of all U.S. senior MD applicants compared to MD-PhD applicants, 2007-2024 (2009-2024 for neurosurgery). P-values from chi-square test showed statistical significance between all MD and MD-PhD applicants match rates only in dermatology. Denominators and sample sizes included in Table 3. Data adopted from NRMP *Charting Outcomes in the Match™,* 2007-2024.

Match outcomes for MD-PhD applicants varied substantially by specialty and year, with no consistent evidence of preferential selection compared to overall U.S. MD seniors (Table 3). The number of MD-PhD applicants has also decreased in this period from 147 applicants across all studied specialties in 2009 to 96 in 2024. In dermatology, MD-PhD applicants consistently achieved statistically significant higher match rates than MD-only applicants from 2007–2024 (p < 0.001, Fig 1). In 2007, 82.9% of MD-PhDs (*n =* 29 of 35) matched compared with 61.2% overall (*n =* 249 of 407), and in 2024 this advantage continued, with 93.3% of MD-PhDs securing a position (*n =* 28 of 30) versus 70.6% of all MD applicants (*n =* 424 of 601) (Table 2).

**Table 3:** Number of U.S. allopathic senior applicants matching into dermatology, neurosurgery, orthopedic surgery, otolaryngology, plastic surgery, and diagnostic radiology, 2007-2024.

Year	Total Matched	% Matched	Total Not Matched	% Not Matched	MD/PHD Matched	% MD/PHD Matched	MD/PHD Not Matched	% MD/PHD Not Matched	95% CI (% of MD-PhD Matched)
Dermatology									
2007	249	61.18%	158	38.82%	29	82.86%	6	17.14%	(67.3%–91.9%)
2009	286	69.59%	125	30.41%	32	94.12%	2	5.88%	(80.9%–98.4%)
2011	307	79.33%	80	20.67%	28	84.85%	5	15.15%	(69.1%–93.3%)
2014	352	76.03%	111	23.97%	17	94.44%	1	5.56%	(74.2%–99.0%)
2016	360	77.09%	107	22.91%	25	92.59%	2	7.41%	(76.6%–97.9%)
2018	368	81.60%	83	18.40%	20	86.96%	3	13.04%	(67.9%–95.5%)
2020	388	84.72%	70	15.28%	35	87.50%	5	12.50%	(73.9%–94.5%)
2022	426	71.60%	169	28.40%	19	76%	6	24%	(56.6%–88.5%)
2024	424	70.55%	177	29.45%	28	93.33%	2	6.67%	(78.7%–98.2%)
Orthopedic Surgery									
2007	577	80.25%	142	19.75%	13	86.67%	2	13.33%	(62.1%–96.3%)
2009	586	78.76%	158	21.24%	15	88.24%	2	11.76%	(65.7%–96.7%)
2011	621	76.95%	186	23.05%	15	83.33%	3	16.67%	(60.8%–94.2%)
2014	648	77.33%	190	22.67%	9	81.82%	2	18.18%	(52.3%–94.9%)
2016	649	75.20%	214	24.80%	12	70.59%	5	29.41%	(46.9%–86.7%)
2018	691	82.36%	148	17.64%	9	75%	3	25%	(46.8%–91.1%)
2020	685	79.65%	175	20.35%	5	83.33%	1	16.67%	(43.6%–97.0%)
2022	703	65.82%	365	34.18%	8	72.73%	3	27.27%	(43.4%–90.3%)
2024	726	73.11%	267	26.89%	7	53.85%	6	46.15%	(29.1%–76.8%)
Neurosurgery									
2007									
2009	171	79.53%	44	20.47%	20	83.33%	4	16.67%	(64.1%–93.3%)
2011	174	86.14%	28	13.86%	18	81.82%	4	18.18%	(61.5%–92.7%)
2014	188	78.99%	50	21.01%	23	88.46%	3	11.54%	(71.0%–96.0%)
2016	200	75.76%	64	24.24%	16	80%	4	20%	(58.4%–91.9%)
2018	203	86.38%	32	13.62%	24	96%	1	4%	(80.5%–99.3%)
2020	203	75.19%	67	24.81% =	18	75%	6	25%	(55.1%–88.0%)
2022	202	74.26%	70	25.74%	19	82.61%	4	17.39%	(62.9%–93.0%)
2024	204	68.69%	93	31.31%	10	66.67%	5	33.33%	(41.7%–84.8%)
Otolaryngology									
2007	249	81.64%	56	18.36%	9	100.00%	0	0.00%	(70.1%–100.0%)
2009	260	79.75%	66	20.25%	7	87.50%	1	12.50%	(52.9%–97.8%)
2011	267	85.58%	45	14.42%	10	90.91%	1	9.09%	(62.3%–98.4%)
2014	277	75.27%	91	24.73%	5	62.50%	3	37.50%	(30.6%–86.3%)
2016	272	88.89%	34	11.11%	8	88.89%	1	11.11%	(56.5%–98.0%)
2018	284	95.62%	13	4.38%	12	100%	0	0%	(75.7%–100.0%)
2020	310	74.88%	104	25.12%	8	80%	2	20%	(49.0%–94.3%)
2022	315	69.23%	140	30.77%	9	100%	0	0%	(70.1%–100.0%)
2024	339	81.88%	75	18.12%	7	77.78%	2	22.22%	(45.3%–93.7%)
Plastic Surgery									
2007	85	62.50%	51	37.50%	3	60.00%	2	40.00%	(23.1%–88.2%)
2009	85	52.47%	77	47.53%	2	22.22%	7	77.78%	(6.3%–54.7%)
2011	74	44.05%	94	55.95%	5	62.50%	3	37.50%	(30.6%–86.3%)
2014	126	70.79%	52	29.21%	5	83.33%	1	16.67%	(43.6%–97.0%)
2016	133	76.88%	40	23.12%	4	66.67%	2	33.33%	(30.0%–90.3%)
2018	156	85.71%	26	14.29%	4	80%	1	20%	(37.6%–96.4%)
2020	165	72.05%	64	27.95%	3	60%	2	40%	(23.1%–88.2%)
2022	173	62.68%	103	37.32%	3	75%	1	25%	(30.1%–95.4%)
2024	188	74.31%	65	25.69%	5	83.33%	1	16.67%	(43.6%–97.0%)
Diagnostic Radiology									
2007	831	90.82%	84	9.18%	40	93.02%	3	6.98%	(81.4%–97.6%)
2009	931	85.73%	155	14.27%	47	85.45%	8	14.55%	(73.8%–92.4%)
2011	883	96.08%	36	3.92%	38	97.44%	1	2.56%	(86.8%–99.5%)
2014	768	98.46%	12	1.54%	33	100%	0	0%	(89.6%–100.0%)
2016	719	97.96%	15	2.04%	30	96.77%	1	3.23%	(83.8%–99.4%)
2018	682	88.92%	85	11.08%	27	90%	3	10%	(74.4%–96.5%)
2020	680	95.51%	32	4.49%	13	100%	0	0%	(77.2%–100.0%)
2022	758	83.21%	153	16.79%	25	86.21%	4	13.79%	(69.4%–94.5%)
2024	777	86.43%	122	13.57%	21	91.30%	2	8.70%	(73.2%–97.6%)

Obtained from NRMP *Charting Outcomes in the Match™*, 2007−2024, Table 1 (Number of Applicants and Positions in the 2011 Main Residency Match by Preferred Specialty) and Chart DM-9 (Other Characteristics of U.S. Seniors). Preferred specialty is defined as the specialty ranked first on an applicant’s rank order list, excluding preliminary programs in specialties except Transitional Year. For applicable specialties, PGY-1 and PGY-2 positions are included. No match data available for neurosurgery in 2007.

In contrast, orthopedic surgery revealed a reversal: although MD-PhD applicants matched at 86.7% in 2007 (*n =* 13 of 15) compared with 80.3% overall (*n =* 577 of 719), their success rate fell to just 53.9% in 2024 (*n =* 7 of 13) as compared to the 73.1% rate among all applicants (*n =* 726 of 993).

Neurosurgery demonstrated fluctuating outcomes, with MD-PhDs initially matching at higher rates (83.3% in 2009 versus 79.5% overall), but by 2024 they matched slightly below their peers (66.7% vs. 68.7%). In otolaryngology, MD-PhD applicants matched at 100% in 2007 (*n =* 9 of 9), but this declined to 77.8% in 2024 (*n =* 7 of 9), while the overall pool remained relatively stable at around 82%. Plastic surgery showed gradual improvement for MD-PhDs, who matched at 60.0% in 2007 (*n =* 3 of 5) compared with 62.5% overall (*n =* 85 of 136), but rose to 83.3% (*n =* 5 of 6) in 2024, surpassing the 74.3% rate for all applicants.

Diagnostic radiology, a specialty with consistently high match rates, showed MD-PhD applicants maintaining a modest but steady advantage, with 93.0% matching in 2007 (*n =* 40 of 43) compared with 90.8% overall (*n =* 831 of 915), and 91.3% in 2024 (*n =* 21 of 23) compared with 86.4% overall (*n =* 777 of 899) (Table 3) [17].

Findings related to compensation, NIH funding, and number of PSTP programs were largely considered exploratory (Table 4). Compensation was not significantly correlated with MD-PhD resident representation (r_s_ = +0.086, p = 0.869) or match advantage (r_s_ = −0.600, p = 0.204). This was driven in part by neurosurgery, which combined the highest mean compensation ($749,100) with the second-highest MD-PhD representation (8.2%). Similarly, total NIH departmental funding showed no meaningful association with representation (r_s_ = −0.029, p = 0.956) or match advantage (r_s_ = +0.200, p = 0.697).

**Table 4:** Specialty-level physician compensation, NIH funding, PSTP availability, and MD-PhD outcomes.

Specialty	Mean Physician Compensation (USD)^a^	Total NIH Funding (USD, 2024)^b^	No. of PSTP Programs^c^	MD-PhD Resident Representation (%, 2014)^d^	Mean MD-PhD Match Advantage (pp, 2007–2024)^e^
Neurosurgery	$763,908	$247,378,877	7	8.2%	+3.62
Orthopedic Surgery	$654,815	$121,667,499	1	1.0%	+0.68
Plastic Surgery	$619,812	$558,758	0	4.9%	−0.93
Diagnostic Radiology	$531,983	$654,115,076	6	2.9%	+1.90
Otolaryngology	$502,543	$173,575,214	1	2.2%	+6.09
Dermatology	$493,659	$100,601,434	12	6.0%	+13.44

^a^Mean physician compensation (USD) in 2024. Specialties ordered from highest to lowest mean physician compensation. Source: Doximity 2024 Physician Compensation Report.

^b^Total NIH awards to departments in each specialty across all funded U.S. medical schools. Source: Blue Ridge Institute for Medical Research (2024).

^c^Number of Physician-Scientist Training Programs (PSTPs) per specialty as listed by the American Physician Scientists Association.

^d^Percentage of active residents holding an MD-PhD degree. Source: 2018 National MD-PhD Program Outcomes Study.

^e^Mean annual difference in match rate (MD-PhD applicants minus all applicants) across available NRMP cycles, 2007–2024. pp = percentage points.

In contrast, the number of PSTP programs demonstrated the strongest associations of any variable. PSTP count was positively correlated with both MD-PhD resident representation (r_s_ = +0.580, p = 0.224) and MD-PhD match advantage (r_s_ = +0.754, p = 0.087). Dermatology, which had the greatest number of dedicated PSTPs (n = 12) among the specialties studied, also demonstrated the highest MD-PhD match advantage (+13.44 pp) and strong MD-PhD representation (6.0%). Conversely, plastic surgery had no identified dedicated PSTPs and showed a negative mean match advantage (−0.93 pp). These findings suggest that the availability of dedicated research training within a specialty may correlate more strongly with MD-PhD match success than compensation or NIH funding.

## Discussion

Research output remains a key factor in residency selection, and medical students are increasingly taking dedicated research years. For instance, among dermatology applicants from 2018 to 2023, 31.3% of applicants reported completing a research year [23]. Yet, despite the research-intensive training inherent to MD-PhD programs and value program directors place on research, our findings show no consistent trend of residency programs preferentially selecting these applicants in competitive specialties [5,18].

While dermatology and diagnostic radiology continue to show relatively strong match outcomes for MD-PhDs, orthopedic surgery and otolaryngology illustrate more variable or even declining success, consistent with prior studies [1]. Taken together, these findings suggest that the value residency programs place on physician-scientist training is heterogeneous and may be outweighed by other selection factors [18,24].

Several explanations may account for these trends, such as the selection effects of residency programs and self-selection of applicants based on perceptions of specialties. On the program side, residency programs may prioritize research volume or specialty-specific research over the quality or depth of research experiences typically achieved during PhD training, reflected in the exponential rise in research deliverables per the latest NRMP report [17]. Because MD-PhD curricula tend to emphasize basic or translational science—which is often more time- and resource-intensive—these applicants may appear less productive in terms of short-term metrics compared with MD-only peers who focus on more rapid, clinically-oriented projects [1]. This difference may inadvertently disadvantage MD-PhDs in specialties that heavily weigh application portfolio size and aligns with the trend where students are choosing to pursue a research year/fellowship to improve output.

On the applicant side, MD-PhD applicants may disproportionately choose specialties perceived as compatible with physician-scientist careers. Prior work has established that MD-PhD graduates gravitate toward specialties offering protected research time, meaning fewer applicants may apply to specialties that do not [15]. Another factor to consider is that many MD-PhD students do not decide on a specialty until after re-entering clinical rotations, underscoring that specialty choices are shaped by more than prior research experience alone. Although limited, training pathways such as the MSTPs, PSTPs, and 2 + 2’s, continue to illustrate the importance of integrating research and clinical training in medicine yet these positions remain exclusive and are infrequently filled by MD-only candidates. MD-PhD graduates who pursue non-clinical career paths after graduation or delay their entrance into residency may also account for some of these findings, as illustrated by the 7.6% of responders to the MD-PhD Outcomes Study indicating they did not pursue postgraduate clinical training [15]. Multiple studies have shown graduates may pursue careers in research, industry, or government rather than pursuing postgraduate residency training [25,26]. Both applicant and programs preferences reinforce one another: applicant self-selection shapes the pool that programs see, while program-level preferences shape which specialties applicants view favorably. Thus, rather than viewing match outcomes as a one-directional preference by programs, match outcomes may better be understood as an ecosystem-level equilibrium between applicants and programs.

As explored in this study, an additional structural factor that may explain variation in MD-PhD representation is the relationship between clinical compensation and NIH-funded salary limitations. As of 2026, the NIH Executive Level II salary cap is $228,000 [27], limiting the proportion of a physician-scientist’s salary that can be covered through federal research funding. In high-compensation specialties, R01 funding covers a small fraction of clinical earning potential, potentially serving as a financial cost to departments that support research-protected time. This push toward clinical productivity may disincentivize these specialties from recruiting or retaining MD-PhD graduates pursuing research-intensive careers, independent of competitiveness [28]. This is consistent with findings in the literature that there is an inverse relationship between earning potential and research effort in different specialties [29]. The role of NIH funding on the MD-PhD match process is well-documented, as trainees in dual-degree programs tend to match at departments with higher NIH funding than MD-only students [30].

Exposure and mentorship play important roles in the physician-scientist pipeline. MD-PhD trainees often lack exemplars within certain specialties who can demonstrate viable physician-scientist career pathways. Without examples or reassurance that a research-focused career is feasible in a given field, applicants may self-select away from that specialty [31]. Efforts by organizations such as the American Physician Scientists Association have sought to address this gap, but the influence of these initiatives on specialty-specific recruitment remains unclear [10]. Furthermore, financial and lifestyle considerations shape specialty choice. MD-PhD graduates endure a prolonged training pathway that delays earning potential, and high-paying specialties tend to have fewer physicians dedicating significant time to research. The opportunity cost of an MD-PhD program, along with limited structural support for protected research time in certain specialties, can steer applicants toward careers emphasizing clinical productivity rather than academic scholarship, [10,29,32] a dynamic that may contribute to the observed variability in representation across fields [29]. Financial pressures on medical institutions and unstable research funding are other factors that may disincentivize MD-PhD graduates from pursuing research careers [12].

Although MD-PhDs account for only a small proportion of medical graduates, they represent more than half of NIH-funded physician investigators. In 2014, over half of physicians funded by the NIH had an MD-PhD, despite research-trained physicians only making up 3% of all medical school graduates [25,29,33]. Their disproportionate role in advancing biomedical research underscores the need to maintain and expand the physician-scientist pipeline across specialties. Residency programs that align selection criteria with the long-term potential of MD-PhD applicants—rather than short-term metrics—may help preserve this segment of the workforce. Specialty exposure, structured mentorship, protected research time, clear academic career pathways, and enhanced financial support are potential strategies to strengthen recruitment and retention [34,35]. Furthermore, given their prolonged training timeline, MD-PhD graduates may remain coveted residency applicants, bringing a level of maturity, scientific rigor, and resilience that distinguishes them from many MD-only peers.

This study has limitations. There may be unmeasured confounding factors that play an important role in residency selection, including program preferences and fit, clinical performance, personal statements, letters of recommendation, diversity initiatives, signaling and geographic preferences [36]. These preferences are not evident in the data in this study. For example, while many programs are focused on producing the best clinicians, others may harbor an interest in training future basic and translational investigators. Favoritism towards an MD-PhD residency applicant will depend on the particular goals of a residency program. The AAMC Report on Residents and the NRMP Charting Outcomes™ datasets lack the granularity to show individual-level research productivity among MD-PhDs. The AAMC Report on Residents, which relies on reporting by registrars, additionally does not discern between graduates of MD-PhD programs and residents who hold an MD and a PhD that were achieved separately. Further, the National MD-PhD Program Outcomes Study relies on self-reported surveys through 2014 with occasional missing values, while the NRMP Charting Outcomes™ dataset similarly had self-reported data.

Future studies may include anonymously, and periodically, surveying program directors of these competitive specialties to better understand the process of ranking and screening applicants with an MD-PhD background. Moving forward, it is critical for residency selection criteria to align with a mission of both clinical care and the valuable insight of physician-scientists. Better understanding the balance between program and applicant selection would require applicant-level data including specialty-specific application rates, interview offer rates, and rank order list for MD-PhD vs. MD-only applicants. Full transparency would help clarify the balance between the applicant and program sides of the match process.

In summary, MD-PhD graduates remain an essential but inconsistently favored applicant group in competitive residency matches. Despite their extensive research training, these applicants are not uniformly advantaged, suggesting that current shifts in residency selection may not fully align with the long-term value physician-scientists bring to medicine. Ultimately, residency programs seek to select clinicians who will provide excellent patient care, and research accomplishments, while valuable, may be secondary to clinical care.

## Supporting information

S1 File MD-PhD Match Data. (XLSX)
